# 10-Shogaol, an Antioxidant from *Zingiber officinale* for Skin Cell Proliferation and Migration Enhancer

**DOI:** 10.3390/ijms13021762

**Published:** 2012-02-08

**Authors:** Chung-Yi Chen, Kuo-Chen Cheng, Andy Y Chang, Ying-Ting Lin, You-Cheng Hseu, Hui-Min Wang

**Affiliations:** 1 Department of Medical Laboratory Science and Biotechnology, School of Medical and Health Sciences, Fooyin University, 151, Ching-Hsueh Road, Ta-Liao District, Kaohsiung 83102, Taiwan; E-Mail: xx377@mail.fy.edu.tw; 2 Department of Internal Medicine, Chi-Mei Medical Centre, Tainan 710, Taiwan; E-Mail: kcg.cheng@gmail.com; 3 Department of Safety Health and Environment, Chung Hwa University of Medical Technology, Tainan 717, Taiwan; 4 Department of Medicine, National Defense Medical Center, Taipei 114, Taiwan; 5 Department of Biology, University of California, Riverside, Riverside, CA 92521, USA E-Mail: Chang.Andy.Y@gmail.com; 6 Department of Fragrance and Cosmetic Science, Kaohsiung Medical University, 100, Shih-Chuan 1st Road, San-Ming District, Kaohsiung 80708, Taiwan; 7 Department of Biotechnology, College of Life Sciences, Kaohsiung Medical University, Kaohsiung 80708, Taiwan; E-Mail: ytlin@kmu.edu.tw; 8 Department of Cosmeceutics, College of Pharmacy, China Medical University, Taichung 404, Taiwan; E-Mail: ychseu@mail.cmu.edu.tw

**Keywords:** ginger, 10-shogaol, antioxidant activities, skin cell, proliferation, growth factors, migration

## Abstract

In this work, one of *Zingiber officinale* components, 10-shogaol, was tested with 1,1-diphenyl-2-picrylhydrazyl (DPPH) radical scavenging, metal chelating ability, and reducing power to show antioxidant activity. 10-Shogaol promoted human normal epidermal keratinocytes and dermal fibroblasts cell growths. 10-Shogaol enhanced growth factor production in transforming growth factor-β (TGF-β), platelet derived growth factor-αβ (PDGF-αβ) and vascular endothelial growth factors (VEGF) of both cells. In the *in vitro* wound healing assay for 12 or 24 h, with 10-shogaol, the fibroblasts and keratinocytes migrated more rapidly than the vehicle control group. Thus, this study substantiates the target compound, 10-shogaol, as an antioxidant for human skin cell growth and a migration enhancer with potential to be a novel wound repair agent.

## 1. Introduction

Antioxidant applications are important for protecting the human body from various sources of oxidative damage and are used extensively for prevention of a variety of diseases. It has many bio-functions including anti-allergenic, anti-inflammatory, anti-bacterial and anti-viral activities, and the prevention of carcinogenesis, diabetes and heart disease [1–5]. Previous studies revealed that oxidative stress has deleterious effects on mesenchymal progenitor cells in terms of decreasing cell proliferation, increasing apoptosis and inhibiting their differentiation [6]. High concentration of free radicals within human body can induce oxidative stress and cellular damage by altering the biological activities of lipids, proteins, DNA and carbohydrates, even to cellular death [7]. In order to protect the human body from various forms of oxidative damage, recently there has been a noticeable increase in the search and identification of natural and safe antioxidants [8]. Dietary supplements and natural antioxidants, from beverages, cereals, fruits, and many vegetables, have gained popularity in recent years due to their abilities to enhance the body’s antioxidant defenses [9]. To decelerate or prevent *in vitro* and *in vivo* oxidation reactions, antioxidants were used to terminate the oxidation chain reactions [10].

Among the many groups of growth factors, TGF-β has been identified to be the most potent growth factor that is able to regulate cell migration, fibrotic scar formation, and wound closure. Furthermore, TGF-β enhances cellular migratory movements, also known as scarring, and stimulates the proliferation of myofibroblast phenotype [11]. It is produced by fibroblasts, keratinocytes, macrophages and platelets. TGF-β, along with other growth factors in the same family, assists in cellular adhesion, differentiation, migration and proliferation. During wound healing procedures, TGF-β is crucial in angiogenesis, connective tissue regeneration, inflammation and re-epithelialization [12]. PDGF-αβ is essential in the proliferation of human skin cells. When the skin is wounded, traces of PDGF-αβ secreted from related cells can be detected in wound fluid. VEGF is exemplified as the vascular permeability factor and is secreted by endothelial, fibroblasts, keratinocytes, macrophages, neutrophils, platelets and smooth muscle cells. VEGF is a vital regulator of endothelial cell migration, proliferation, and permeability in physiological and pathological angiogenesis [13]. In extracellular matrix (ECM) development, PDGF-αβ and TGF-β induce the permeation and the conversion of fibroblasts to myofibroblasts which creates the constrictive forces that helps the closure of the wound. PDGF-αβ secretes TGF-β by stimulating immune macrophages. PDGF-αβ also cooperates synergistically with hypoxia ischemic damage tissues to increase the VEGF expression [14]. All the growth factors mentioned have interactions with each other during skin cell proliferations or wound healing processes.

**Figure 1:**
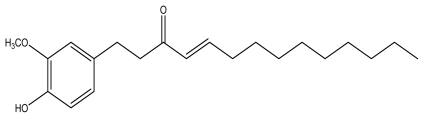
The structure of ginger compound, 10-shogaol.

Ginger, the powdered rhizomes of the herb *Zingiber officinale* Roscoe (Zingiberaceae), is a spice that is widely used in many types of cuisine. In traditional Chinese medicine, ginger, known to be a cure-all remedy, is used in treatments for ailments such as allergy, anti-microorganism infection, asthma, constipation, diabetes, gingivitis, nervous diseases, rheumatism, and stroke [15]. Shogaols are compounds formed by dehydrating gingerols, and also were not thought to be present in fresh rhizome (Figure 1). Typical proportions of 6-, 8- and 10-gingerol are 58%, 22% and 20%, respectively. The ratios of 6-, 8- and 10-shogaols depends on the extent of the dehydration process and storage of gingerols [16,17]. 6-Shogaol was described to not only have anti-cancer and anti-inflammatory capabilities, but also had the potential to be used as an anti-metastatic treatment [18]. At pro-apoptotic concentrations, 10-shogaol, an extract from ginger, was able to induce G(2)/M arrest and abnormal mitotic cell death that is associated with tubulin aggregation [19]. 10-Shogaol, the only non-pungent compound among the gingerols and shogaols, has the ability to stimulate the increase of adrenaline secretion [20]. Literature studies showed that the multiplication of free radicals in cells resulted in the suppression of cell viability, which implies that antioxidant exhibits relatively positive effects on cell proliferation. It was demonstrated that free radicals inhibit the proliferation and migration of vascular smooth muscle cells, both *in vivo* and *in vitro*, that contributes to vascular injury [21]. In this study, antioxidant activities were explored by employing various established *in vitro* systems. There were several *in vitro* and *in vivo* reports about the skin cell proliferation induced by curcumin and ginger extracts [22,23]. The goal of this study was to systematically evaluate 10-shogaol’s ability to enhance human normal skin cell growth. Furthermore, this was also the first attempt to demonstrate the bio-activites of 10-shogaol for medical cosmetology wound repair purposes.

## 2. Results and Discussion

### 2.1. Antioxidant Activities

Antioxidants have singlet oxygen-quenching properties, free radical-scavenging abilities and transition metal-chelating capacities. Through the use of 1,1-diphenyl-2-picrylhydrazyl (DPPH) radical scavenging method, metal chelating ability test and reducing power assay antioxidant properties in ginger compound, 10-shogaol, was tested. The data is expressed as a mean value of three independent experiments with ± standard deviation (SD). In DPPH free radical scavenging assay, 10-shogaol inhibits the oxidative products which allows it to form a stable complex (Table 1). In the assay, 10-shogaol reduced the stable radical DPPH to a yellow-colored diphenyl-picrylhydrazine. The results showed that 10-shogaol has middle to high inhibitory effect (34.54%) in comparison to vitamin C which has high inhibitory effect (92.02%).

Ferrozine quantitatively formed complexes with Fe^2+^. With the existence of chelating agents, such as 10-shogaol, the complex becomes disrupted resulting in the appearance of a bright red color. The inhibition of 10-shogaol was <10.00% with a minor level of Fe^2+^ scavenging effect, whereas ethylene diamine tetra-acetic acid (EDTA) presented a strong scavenging ability with a 84.78% inhibition (Table 1).

In the reducing power assay, the color of the testing solutions changes from yellow to different shades between green and dark blue depending on the reducing power of the antioxidant. The presence of 10-shogaol is similar to the antioxidant substances in the antioxidant samples that induces the reduction of Fe^3+^/ferricyanide complex to the ferrous form. As seen in Table 1, 10-shogaol is shown to have a moderate reducing power at 100 μM (OD_700_ = 0.60) while 3-*tert*-butyl-4-hydroxyanisole (BHA) at the same dosage level (OD_700_ = 0.94).

Antioxidants are molecules that decelerate and prevent the oxidation reaction *in vitro* and *in vivo* by terminating the oxidation chain reaction. Numerous crude extracts and pure natural compounds from plants are reported to have antioxidant and radical scavenging activities [10]. Over accumulation and formation of free radicals would accelerate the oxidation of lipids, found in cosmetics and foods with low quality, which will result in a reduced consumer acceptance [24]. Overall, 10-shogaol showed moderate antioxidant activities with the application in pharmacology to improve current treatments for diseases.

### 2.2. Cell Viabilities

**Figure 2:**
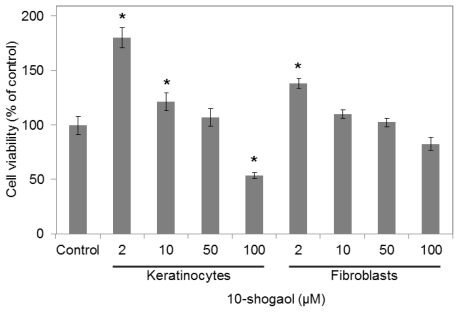
Human keratinocytes and fibroblasts cell growths were treated with 10-shogaol at various concentrations for 24 h culture. The graph illustrates the mean ± SD of three independent experiments. Bars: SD. * *p* < 0.05 against the vehicle control group.

10-Shogaol was used on human skin cells to examine its effects on skin cell viability. [3-(4,5-Dimethylthiazol-2-yl)-2,5-diphenyltetrazolium bromide] (MTT) assay was performed to test if 10-shogaol has cytotoxic properties on keratinocytes (epidermal) and fibroblasts (dermal) (Figure 2). Various concentrations of 10-shogaol were added to both keratinocytes and fibroblasts, in order to observe their effect on cell proliferation. In keratinocytes, 2 μM displayed the highest increase in cell viability (180.25%) compared to the vehicle group. No significant difference was observed in concentration levels 50 μM. The cell viabilities gradually decreased in a dose-dependent manner from 2 to 100 μM. There was a discrepancy between this study and our previous ones, where ginger compounds were discovered for their anticancer properties in inhibiting cell growth and migration [25,26]. It is assumed that the dosages, treatment periods or cell types in the different experimental designs are the cause of the different cell growth.

Similar to keratinocytes, fibroblasts also demonstrated a high increase in cell proliferation with the addition of 2 μM of 10-shogaol (138.01%). There was also no noticeable difference when 10 and 50 μM of 10-shogaol was added to fibroblast. 10-Shogaol, at low doses of 2 μM, has shown to have positive effects on cell viability along with increasing the rate of cell proliferation in both keratinocytes and fibroblasts.

### 2.3. Increasing Growth Factor Production (TGF-β, PDGF-αβ and VEGF) of Human Fibroblasts and Keratinocytes by 10-Shogaol

**Figure 3:**
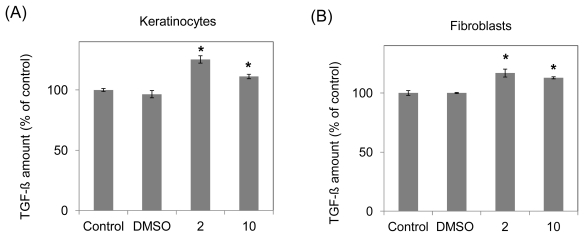

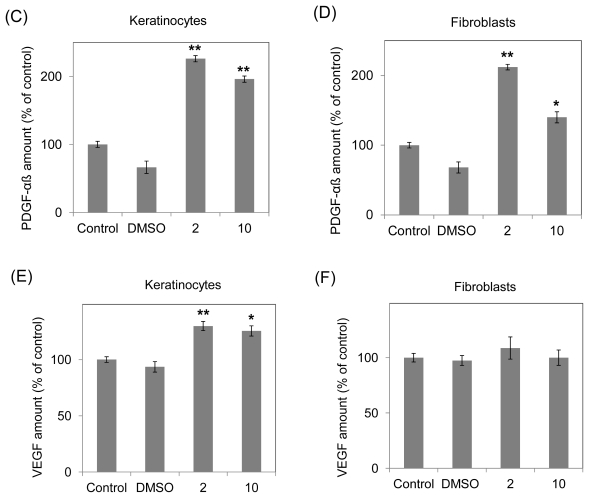
Three growth factors secretive productions effected by 10-shogaol on human skin keratinocytes (**A**,**C**,**E**) and fibroblasts (**B**,**D**,**F**). The data was shown as mean ± SD of three independent experiments. Significance for three different time-point groups was accepted at * *p* < 0.05 or ** *p* < 0.01 *versus* their corresponding controls.

TGF-β, PDGF-αβ and VEGF, growth factors that are renowned cell proliferation enhancers have been chosen to be evaluated. As shown in Figure 3, the expression of growth factors was observed using enzyme linked immunosorbent assay (ELISA). To ensure that dimethyl sulfoxide (DMSO) does not affect the assays, DMSO is comprised of 1% of the comparing control. No significant difference was observed between assays with DMSO or without. The results showed that TGF-β in keratinocytes increased from 665.4 to 834.2 pg/mL (125.37%) and 740.0 pg/mL (111.21%) after the incubation of 10-shogaol at 2 and 10 μM for 24 h, respectively (Figure 3A). In fibroblasts, TGF-β increased from 778.3 to 909.5 pg/mL (116.9%) and 878.3 pg/mL (112.9%) after we incubated the compound at 2 and 10 μM for 24 h, respectively as shown in Figure 3B. For PDGF-αβ in keratinocytes, the concentration content varied obviously. 10-Shogaol treatments at 2 μM showed the greatest increase in concentration values (2.20 to 4.97 pg/mL, 225.9%), followed by 10-shogaol treatments at 10 μM (2.20 to 4.31 pg/mL, 195.9%) in Figure 3C. In Figure 3D, 10-shogaol treatments at 2 μM also presented the greatest increase in concentration values (2.50 to 5.32 pg/ml, 212.8%), followed by 10-shogaol treatments at 10 μM (2.5 to 3.55 pg/mL, 142.0%) of fibroblasts. For VEGF in keratinocytes, 10-shogaol treatments at 2 μM demonstrated the increase in concentration values (250.7 to 325.5 pg/mL, 129.8%), followed by 10-shogaol treatments at 10 μM (250.7 to 314.5 pg/mL, 125.4%) in Figure 3E. In fibroblasts, 10-shogaol treatments at 2 μM showed an increase of 108.8% (287.1 to 312.3 pg/mL). 10-Shogaol treatments at 10 μM illustrated no considerable difference when compared to the vehicle group (Figure 3F).

The formation of granulation by TGF-β provides the protection to the healthy tissues that surrounds the wounded area. TGF-β, with the separation of de-granulating platelets at the wound site, has an impact on the angiogenesis, ECM deposition, inflammatory response, re-epithelialization, and remodeling [27]. With the addition of 10-shogaol, both keratinocytes and fibroblasts’ TGF-β concentration rose significantly in comparison with the vehicle group. TGF-β level was slightly higher in keratinocytes than in fibroblast (Figure 3A,B). PDGF-αβ is generated expressed and secreted by fibroblasts, keratinocytes, macrophages, platelets and vascular endothelium. By recruiting pericytes and strengthening its structural foundation, PDGF-αα, during wound repairing, plays a predominant role in blood vessels development [28]. When treated with 10-shogaol, PDGF-αβ concentration level was noticeably higher in keratinocytes than in fibroblasts in Figure 3C,D. 10-Shogaol was able to increase the PDGF-αβ and TGF-β concentration allowing it to have skin injury repair applications. In wound healing, VEGF, a remarkable growth factor, expedites skin injury repair by stimulating angiogenesis, cell proliferations and migrations [29]. VEGF assists in the organization and synthesis of ECM, which is essential to the restoration of ECM near the wounded tissue zone. After the treatment with 10-shogaol, there were visible increases of VEGF concentrations in keratinocytes and fibroblasts in Figure 3E,F. The increase in keratinocytes was especially significant. In our assumptions, the VEGF production was not up-regulated by the 10-shogaol in fibroblasts. As stated in the introduction, upon injury, the stimulation of immune macrophages by PDGF-αβ triggers immune macrophages to secrete TGF-β, which also aids in the rise of VEGF expression. We advocated that the increase of TGF-β, PDGF-αβ and VEGF regulatory secretions might be due to the addition of 10-shogaol.

### 2.4. Cell Migrations of Fibroblasts and Keratinocytes Enhanced by 10-Shogaol

**Figure 4:**
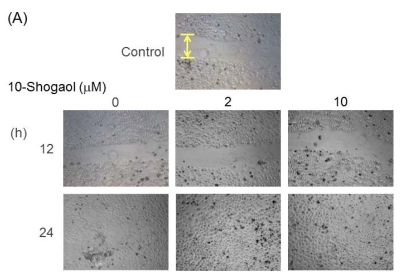

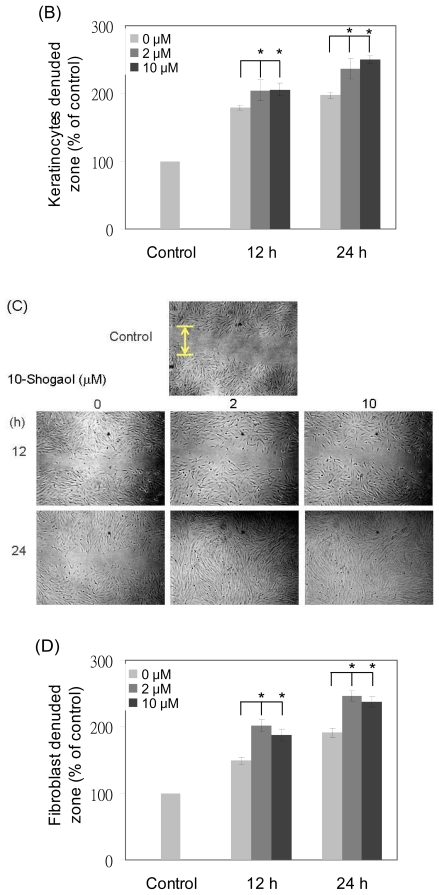
*In vitro* wound healing assay on 10-shogaol effects on human skin cells. (**A**) Migratory photographs of human keratinocytes; (**B**) Quantification of the migration potential of 10-shogaol- or PBS-treated cells; (**C**) Photographs of fibroblasts; (**D**) Fibroblasts migration quantifications. The data was shown as mean ± SD of three independent experiments. Significance for three different time-point groups was accepted at * *p* < 0.05 *versus* their corresponding controls.

To determine the effects of 10-shogaol on keratinocytes and fibroblasts migration, an *in vitro* wound healing assay was performed (Figure 4). The keratinocytes and fibroblasts migration rates were based on the efficiency of monolayer cells invading the wound region with the 10-shogaol treatments at 2 and 10 μM in 12 and 24 h testing periods. Keratinocytes and fibroblasts were individually cultured to monolayer confluence on an uncoated 6-well culture dish followed by applying a scratch with a sterile pipette tip. After extensive washing with PBS, cells were treated with either vehicle or varying concentrations of 10-shogaol for the indicated time periods. The photos of cell repairing enhancing effects of 10-shogaol on keratinocytes migrations can be observed in Figure 4A. Quantification analysis of the increasing abilities of 10-shogaol in treatment conditions are shown in Figure 4B. Keratinocytes exhibited an increase in cell migration with the addition of 10-shogaol. At 2 μM, the cell migration exhibited a greater migration rate at 24 h (236.0%) than at 12 h (204.76%). At 10 μM, the cell migration displayed a higher migration rate at 24 h (250.44%) than at 12 h (205.65%). The treatments of 10-shogaol in fibroblasts also showed the rise in cell migrations. When treated with 2 μM, the cell migration was 201.67% at 12 h and 246.06% at 24 h. When cured with 10 μM, the cell migration was 187.82% at 12 h and 237.92% at 24 h.

Ginger compounds have the ability to induce faster abrasion wound repair by decreasing reactive oxygen species or modulating collagen [30]. The assay system that was used to test the effects of 10-shogaol on keratinocytes and fibroblasts migration abilities was cultured from normal human skin cells. Skin wound healing was slower as opposed to those that were treated with ginger compounds, which induced the skin to repair much more efficiently [23]. In the *in vitro* wound repairing assay, 10-shogaol treated-cells migrated to the clearing area at a faster pace and rate than the control group. When treated with our target compound for 24 h, the wound region was almost completely covered by keratinocytes and fibroblast migratory cells.

In the 12 h 10-shogaol (2 μM) treatment of keratinocytes, the pre-wound region appeared narrower than the vehicle control which had a larger gap. After 24 h, the treated wound area was completely closed and fully proliferated as opposed to the control which still had a gap. On the other hand, at 10 μM treatment of 10-shogaol on keratinocytes, the wound space seemed to be nearly closed while the control’s opening appears the same as when it started. At 24 h, the treated area was entirely closed unlike the control which still had some openings. When fibroblasts was treated with 10-shogaol (2 μM) for 12 h, the injured region appeared to be thinner and the cell proliferation rate seemed to increase while the controlled region had no visible changes. At 24 h, the wound area, treated with 10-shogaol (2 μM), was fully closed unlike the control which still had a gap. When fibroblasts were treated with 10-shogaol (10 μM) for 12 h, the injured area appeared to be closing and the cells were migrating toward the middle. At 24 h, the treated wound region was completely repaired while the control was healing at a slower rate. In this section, it is noticeable that 10-shogaol induced the expression of three growth factors which stimulated the migration and proliferation of keratinocytes and fibroblasts. In this study, it was suggested that 10-shogaol enhanced skin cell growth and migration.

## 3. Experimental Section

### 3.1. Materials

DMSO, DPPH, vitamin C, EDTA, BHA, potassium ferricyanide [K_3_Fe(CN)_6_], trichloroacetic acid, FeCl_3_, FeCl_2_·4H_2_O, MTT, penicillin, streptomycin, and amphotericin B were purchased from Sigma-Aldrich (St. Louis, MO, USA). Fetal bovine serum (FBS) and Dulbecco’s modified Eagle’s medium (DMEM) was obtained from GIBCO BRL (Gaithersburg, MD, USA). All other chemical buffers and reagents were purchased at the highest commercial purity and quality possible.

### 3.2. Plant Materials

Dried and chipped rhizomes (25.6 kg) of *Z. officinale* were extracted repeatedly with different mixtures of chloroform (50 L × 4) at room temperature, based on previous reports [31]. The combined chloroform extracts (896.5 g) were then evaporated further and separated into 20 fractions by column chromatography (CC) in the silica gel (3.8 kg, 70–230 mesh) with gradients of *n*-hexane/CHCl_3_. Fr. 9 (121.2 g), eluted with CHCl_3_-MeOH (50:1), was next subjected to silica gel CC (CHCl_3_-MeOH mixtures) and yielded 10-shogaol (210 mg). Fr. 10 (86.9 g), eluted with CHCl_3_-MeOH (50:1), was next repeatedly subjected to silica gel CC (CHCl_3_-MeOH mixtures) and yielded 6-shogaol (328 mg). The extraction yielded pure 10-shogaol from different fractions by silica gel column chromatography with gradients of *n*-hexane/CHCl_3_ (Figure 1). Using spectroscopy, the compound was analyzed and identified, and compared with literature values [32]. The purity of 10-shogaol was >95% as determined by HPLC. The chemical structure of 10-shogaol was confirmed by NMR [32].

### 3.3. Determination of DPPH Radical Scavenging Capacity

The antioxidant activity of the target compound, 10-shogaol, was measured in terms of hydrogen donating or radical scavenging ability using the modified DPPH method [33]. Different concentrations of the samples were added to 0.2 mL of a DPPH (60 μM) solution. When DPPH reacts with an antioxidant compound that donates hydrogen, it is reduced resulting in a decrease in the absorbance at 520 nm. The absorbance was recorded at 5 min intervals for 30 min using a UV visible spectrophotometer and was evaluated at the end point (30 min). Vitamin C was used as a positive control. The percentages of remaining DPPH were plotted against the sample to obtain the amount of antioxidant required to reduce the initial concentration of DPPH. Scavenging activity (%) was determined with the following equation:

(1) $$100\times ({OD}_{\text{control}}-{OD}_{\text{sample}})/{OD}_{\text{control}}$$

### 3.4. Metal Chelating Activity

The ferrous ion chelating potential of chlorophyll was investigated according to a previously described method [33]. Briefly, various testing concentrations of samples dissolved in DMSO were added to a solution of 2 mM FeCl_2_·4H_2_O (0.05 mL). The reaction was initiated by the addition of 5 mM ferrozine (0.2 mL), and the mixture was vigorously shaken and left standing at room temperature for 10 min. The absorbance of the mixture was then read at 560 nm against a blank. EDTA was used as a positive control, and the formula for calculation of the chelating activity was similar to Equation (1).

### 3.5. Reducing Power Assay

The reducing power of 10-shogaol was determined according to a previously described method [33]. Various concentrations of testing samples in 0.063 mL of methyl alcohol were mixed with 0.1 mL of 0.2 M sodium phosphate buffer (pH 6.8) and 2.5 μL of 20% potassium ferricyanide (K_3_Fe(CN)_6_). The mixture was incubated at 50 °C for 20 min, and 0.16 mL of trichloroacetic acid (10%) was added to the mixture that was then centrifuged for 10 min at 3000 g. The upper layer of the solution (75 μL) was mixed with distilled water (25 μL) and 2% FeCl_3_ (25 μL), and the absorbance was measured with a 96-well plate spectrophotometer at 650 nm. BHA was used as a positive control. A higher absorbance demonstrates a higher reductive capability.

### 3.6. Human Dermal Fibroblasts and Epidermal Keratinocytes Cultures

Both human skin cell culture procedures followed the process of [34]. Briefly, the primary cultures of human skin fibroblasts, derived from Chung-Ho Memorial Hospital, Kaohsiung Medical University, Taiwan, ROC (KMUH-IRB-990269); were incubated in Eagle’s medium (DMEM) with 10% fetal calf serum, 100 μg/mL penicillin, 100 μg/mL streptomycin, and 250 ng/mL amphotericin B. Human keratinocytes were isolated from foreskin primary culture and cultured in Keratinocyte-SFM (10724; GIBCO^TM^), supplemented with Bovine Pituitary Extract (BPE, cat. # 13028-014), and EGF (cat. # 10450-013). The medium and growth supplements for keratinocytes contain γ-epidermal growth factor, BPE, insulin, fibroblast growth factor and calcium (0.09 mM). All cell types were incubated at 37 °C in a humidified incubator 5% CO_2_ atmosphere.

### 3.7. Cell Growth Assay

The MTT assay was used to measure cell growth to test the compound used in this study could induce cell proliferation [35]. Briefly, skin cells were seeded in 96-well plates and treated with different concentrations of ginger compound or untreated (as positive control) for 24 h. Stock MTT solution (5 mg/mL, dissolved in phosphate buffered saline, PBS) was diluted 1:10 in culture medium and added to a culture dish, then incubated at 37 °C for 2 h. At the end of the incubation period, the medium was removed and replaced with 0.05 mL DMSO to dissolve the formazan crystals. The culture dishes were gently shaken for 20 min in the dark and added to a 96-well plate reading at 595 nm on a multiwall scanning spectrophotometer (UV-vis, BioTek, USA). The cell growth was calculated by the percentage of the control OD (595 nm) value (UV-vis, BioTek, USA). In consideration of the possible antiproliferative effects of DMSO, cultures were added with maximal 1% DMSO and used as positive controls, which was not found to affect skin cell growth.

### 3.8. ELISA Assays

We performed ELISA to determine the amounts of TGF-β, VEGF, and PDGF produced in skin cells after being exposed to the testing compound. The skin cells were cultured in 6-well plates under conditioned medium and the supernatant was collected at 24 h for analysis. Amounts of TGF-β, VEGF and PDGF-αβ secreted in the culture medium were determined from DuoSet ELISA development kits (R & D Systems, USA). The assay was performed according to the manufacturer’s instructions, and protein amounts were measured and given as pictogram per milliliter with standard deviations (SD) of data derived from duplicate measurements.

### 3.9. *In Vitro* Wound Healing Assay

The potential of cellular migration was determined by wound healing migration assays, which was performed according to the methods reported by [3,35]. In brief, 5 × 10^5^ cells were cultured in 12-well plates, and grown to complete confluence. A yellow 200 μL plastic pipette tip was used to create a clean 1-mm-wide wound area on a confluent culture of skin cells and washed three times to remove floating cells. Then, it was added with either vehicle (medium containing 0.5% DMSO) or various concentrations of testing samples for the indicated time periods. After the indicated incubation time, the wound gaps were photographed using an inverted phase-contrast microscopy (TE2000-U; Nikon, Tokyo, Japan) equipped with NIS-Elements (Nikon) Software. The migration and cell movement throughout the wound area were examined and calculated by the free software “TScratch” (www.cse-lab.ethz.ch/software.html) [36]. Magnification: 100× Bars, SD.

### 3.10. Statistical Analysis

All data values are presented as the mean values (±SD) of at least three independent experiments. Where appropriate, data were analyzed by the Student’s *t* test.

## 4. Conclusions

We identified one compound from *Z. officinale*, 10-shogaol, which has the ability to promote normal human skin cell growth (epidermal keratinocytes and dermal fibroblasts). In the cell viability tests, cell proliferation capabilities decreased from low to high dose concentration and the cell growth was greatest at 2 μM. In the *in vitro* wound healing assay, 10-shogaol, at 12 h and 24 h, enhanced fibroblasts and keratinocytes migration. 10-Shogaol-treated fibroblasts and keratinocytes showed higher growth factor productions in transforming growth factor-β (TGF-β), platelet derived growth factor-αβ (PDGF-αβ) and vascular endothelial growth factors (VEGF). Thus, this work provided the molecular basis of 10-shogaol as a novel potential wound repairing agent.

## Figures and Tables

**Table 1:** Antioxidant effects of 10-shogaol.

Samples	DPPH. Scavenging (%)	Metal Chelating (%)	Reducing Power (100 μM, OD_700_)
10-Shogaol	34.54 ± 0.02	<10.00	0.60 ± 0.02
Vitamin C a	90.02 ± 0.40	-	-
EDTA b	-	94.78 ± 0.60	-
BHA c	-	-	0.98 ± 0.11

a Vitamin C was used as a positive control on DPPH assay;

b EDTA was used as a positive control on metal chelating ability at 100 μM;

c BHA was used as a positive control on reducing power at 100 μM, and so did testing samples;

-: no testing.

## Abbreviations

BHA: 3-*tert*-butyl-4-hydroxyanisole
DMEM: Dulbecco’s modified Eagle’s medium
DMSO: dimethyl sulfoxide
DPPH: 1,1-diphenyl-2-picrylhydrazyl
EDTA: ethylene diamine tetra-acetic acid
FBS: fetal bovine serum
PDGF-αβ: platelet derived growth factor-αβ
K_3_Fe(CN)_6_: potassium ferricyanide
TGF-β: transforming growth factor-β
VEGF: vascular endothelial growth factors
